# Supplementary material for: Analysis of PPARγ Signaling Activity in Psoriasis
Source: Int J Mol Sci. 2021 Aug 10;22(16):8603. doi: 10.3390/ijms22168603 (PMC8395241; doi:10.3390/ijms22168603)
Supplement: Supplementary file 1 [file ijms-22-08603-s001.zip › Supplemental materials_Analysis of PPARg signaling activity in psoriasis/Pathway models/Models images and html files/Interleukin-17 and interleukin-22 signaling in psoriasis/64783.html]

IL17RC


# Protein IL17RC

|  |  |
| --- | --- |
| URN | urn:agi-llid:84818 |
| Total Entities | 0 |
| Connectivity | 250 |
| Name | IL17RC |
| Description | interleukin 17 receptor C |
| Notes | This gene encodes a single-pass type I membrane protein that shares similarity with the interleukin-17 receptor (IL-17RA). Unlike IL-17RA, which is predominantly expressed in hemopoietic cells, and binds with high affinity to only IL-17A, this protein is expressed in nonhemopoietic tissues, and binds both IL-17A and IL-17F with similar affinities. The proinflammatory cytokines, IL-17A and IL-17F, have been implicated in the progression of inflammatory and autoimmune diseases. Multiple alternatively spliced transcript variants encoding different isoforms have been detected for this gene, and it has been proposed that soluble, secreted proteins lacking transmembrane and intracellular domains may function as extracellular antagonists to cytokine signaling. [provided by RefSeq, Feb 2011] |
| Primary Cell Localization | Plasma membrane |
| Class | Receptor |

---

|  |  |
| --- | --- |
| Pathway | IL17 Signaling in Psoriasis |
|  | Il17 Signaling Related Neutrophilia in Asthma |
|  | IL17F Signaling in Bronchial Epithelial Cell in Asthma |
|  | Synovial Fibroblast Activation in Psoriatic Arthritis |
|  | Keratinocyte Activation in Psoriatic Arthritis |
|  | Proteins Involved in Endometriosis |
|  | Cytokines Trigger Otitis Media |
|  | 2\_Interleukin-17 and interleukin-22 signaling in psoriasis |
|  | 3\_2\_Regulation of airway surface liquid (ASL) thickness |
|  | Interleukin-17 and interleukin-22 signaling in psoriasis |

---

|  |  |
| --- | --- |
| MedScan ID | 84818 |

---

|  |  |
| --- | --- |
| LocusLink ID | 84818 |
|  | 171095 |
|  | 297520 |

---

|  |  |
| --- | --- |
| Alias | Gm19850 |
|  | FLJ95963 |
|  | IL17F receptor |
|  | interleukin 17 receptor-like |
|  | 1110025H02Rik |
|  | interleukin 17 receptor C |
|  | OTTMUSP00000028724 |
|  | zcytoR14 |
|  | OTTHUMP00000180659 |
|  | RIKEN cDNA 1110025H02 gene |
|  | interleukin IL17 receptor-like |
|  | interleukin IL17F receptor |
|  | LOC100503711 |
|  | UNQ6118/PRO20040/PRO38901 |
|  | interleukin 17F receptor |
|  | OTTMUSP00000028725 |
|  | MGC10763 |
|  | interleukin IL17 receptor C |
|  | OTTHUMP00000207644 |
|  | IL17RC |
|  | OTTMUSP00000028723 |
|  | CANDF9 |
|  | IL17rl |
|  | IL-17 receptor C |
|  | FLJ96005 |
|  | interleukin IL17RC |
|  | interleukin IL17rl |
|  | 1110025H02 |
|  | OTTHUMP00000207646 |
|  | IL17-RL |
|  | IL-17RL |
|  | IL17Rhom |
|  | interleukin-17 receptor homolog |
|  | interleukin-17 receptor-like protein |
|  | interleukin-17 receptor C |
|  | IL-17RC |
|  | IL17-RC |

---

|  |  |
| --- | --- |
| GO ID | 0030368 |
|  | 0005102 |
|  | 0050832 |
|  | 0071621 |
|  | 0097400 |
|  | 1900017 |
|  | 2000778 |
|  | 0009986 |
|  | 0016021 |
|  | 0005886 |
|  | 0016020 |
|  | 0019221 |
|  | 0005887 |

---

|  |  |
| --- | --- |
| KEGG ID | hsa:84818 |
|  | mmu:171095 |

---

|  |  |
| --- | --- |
| Organism | Homo sapiens {Organism urn:agi-taxid:9606} |
|  | Mus musculus {Organism urn:agi-taxid:10090} |
|  | Rattus norvegicus {Organism urn:agi-taxid:10116} |
|  | Homo sapiens |
|  | Mus musculus |
|  | Rattus norvegicus |

---

|  |  |
| --- | --- |
| Mouse chromosome position | 6 |

---

|  |  |
| --- | --- |
| OMIM ID | 610925 |
|  | 616445 |

---

|  |  |
| --- | --- |
| Rat chromosome position | 4q42 |

---

|  |  |
| --- | --- |
| Hugo ID | 18358 |

---

|  |  |
| --- | --- |
| Human chromosome position | 3p25.3 |

---

|  |  |
| --- | --- |
| Swiss-Prot Accession | Q8NAC3 |
|  | Q8NAC3.2 |
|  | Q8BPI5 |
|  | Q8K4C2 |
|  | Q8K4C2.3 |
|  | D3ZIM0 |
|  | A8BWC1 |
|  | A8BWC9 |
|  | A8BWD5 |
|  | E9PHG1 |
|  | E9PHJ6 |
|  | Q6UVY3 |
|  | Q6UWD4 |
|  | Q8NFS1 |
|  | Q9BR97 |
|  | G3X9C0 |
|  | Q99J43 |

---

|  |  |
| --- | --- |
| GenBank ID | NC\_000003 |
|  | NR\_037807 |
|  | NM\_001203264 |
|  | NP\_001190193 |
|  | XM\_017007327 |
|  | XP\_016862816 |
|  | XM\_017007329 |
|  | XP\_016862818 |
|  | XM\_017007336 |
|  | XP\_016862825 |
|  | XM\_024453793 |
|  | XP\_024309561 |
|  | XM\_017007339 |
|  | XP\_016862828 |
|  | XM\_017007338 |
|  | XP\_016862827 |
|  | XM\_017007326 |
|  | XP\_016862815 |
|  | NM\_153461 |
|  | NP\_703191 |
|  | XM\_017007333 |
|  | XP\_016862822 |
|  | XM\_017007328 |
|  | XP\_016862817 |
|  | XM\_017007330 |
|  | XP\_016862819 |
|  | XM\_017007331 |
|  | XP\_016862820 |
|  | XM\_017007332 |
|  | XP\_016862821 |
|  | XM\_017007335 |
|  | XP\_016862824 |
|  | XM\_024453794 |
|  | XP\_024309562 |
|  | XM\_017007334 |
|  | XP\_016862823 |
|  | XM\_017007337 |
|  | XP\_016862826 |
|  | XM\_017007340 |
|  | XP\_016862829 |
|  | NM\_153460 |
|  | NP\_703190 |
|  | NM\_001203263 |
|  | NP\_001190192 |
|  | NM\_001367278 |
|  | NP\_001354207 |
|  | XM\_017007341 |
|  | XP\_016862830 |
|  | NM\_032732 |
|  | NP\_116121 |
|  | NM\_001367280 |
|  | NP\_001354209 |
|  | NM\_001203265 |
|  | NP\_001190194 |
|  | XM\_017007345 |
|  | XP\_016862834 |
|  | XM\_017007348 |
|  | XP\_016862837 |
|  | XM\_017007342 |
|  | XP\_016862831 |
|  | XM\_017007343 |
|  | XP\_016862832 |
|  | XM\_017007347 |
|  | XP\_016862836 |
|  | NM\_001367279 |
|  | NP\_001354208 |
|  | NG\_029376 |
|  | AC018809 |
|  | CH471055 |
|  | EAW64021 |
|  | EAW64022 |
|  | EAW64023 |
|  | EAW64024 |
|  | EAW64025 |
|  | EAW64026 |
|  | EAW64027 |
|  | EAW64028 |
|  | EAW64029 |
|  | EAW64030 |
|  | EAW64031 |
|  | EAW64032 |
|  | CS399136 |
|  | CAL44891 |
|  | CS399138 |
|  | CAL44892 |
|  | GM832015 |
|  | CAV28583 |
|  | GM832017 |
|  | CAV28584 |
|  | GM832034 |
|  | CAV28588 |
|  | JA381717 |
|  | CCB07365 |
|  | JA381719 |
|  | CCB07366 |
|  | KF457576 |
|  | AF458065 |
|  | AAM77569 |
|  | AK075534 |
|  | BAC11677 |
|  | AK092907 |
|  | BAC04001 |
|  | AK094746 |
|  | AK096824 |
|  | AK225650 |
|  | AK290670 |
|  | BAF83359 |
|  | AK294616 |
|  | BAG57799 |
|  | AK295377 |
|  | BAG58336 |
|  | AK303171 |
|  | BAG64270 |
|  | AK308241 |
|  | AK315024 |
|  | AK315053 |
|  | AY358840 |
|  | AAQ89199 |
|  | AY359098 |
|  | AAQ89456 |
|  | BC006411 |
|  | DC381087 |
|  | DC386743 |
|  | EF676032 |
|  | ABV44615 |
|  | EF676033 |
|  | ABV44616 |
|  | EF676034 |
|  | ABV44617 |
|  | Q8NAC3 |
|  | NC\_000072 |
|  | NM\_178942 |
|  | NP\_849273 |
|  | NM\_134159 |
|  | NP\_598920 |
|  | XM\_006505665 |
|  | XP\_006505728 |
|  | XM\_006505666 |
|  | XP\_006505729 |
|  | XR\_001785101 |
|  | XM\_006505668 |
|  | XP\_006505731 |
|  | XM\_006505667 |
|  | XP\_006505730 |
|  | AC153910 |
|  | CH466523 |
|  | EDK99476 |
|  | EDK99477 |
|  | EDK99478 |
|  | CS135270 |
|  | CAJ21523 |
|  | AF458066 |
|  | AAM77570 |
|  | AI463827 |
|  | AI592368 |
|  | AK033890 |
|  | AK040950 |
|  | AK075634 |
|  | BAC35870 |
|  | AK209010 |
|  | AW045329 |
|  | BC004759 |
|  | AAH04759 |
|  | BQ551040 |
|  | BY018830 |
|  | Q8K4C2 |
|  | NC\_005103 |
|  | XM\_017592574 |
|  | XP\_017448063 |
|  | XM\_017592575 |
|  | XP\_017448064 |
|  | XM\_017592576 |
|  | XP\_017448065 |
|  | XM\_017592577 |
|  | XP\_017448066 |
|  | XM\_017592578 |
|  | XP\_017448067 |
|  | XM\_017592579 |
|  | XP\_017448068 |
|  | NM\_001170565 |
|  | NP\_001164036 |
|  | XM\_017592581 |
|  | XP\_017448070 |
|  | XM\_017592580 |
|  | XP\_017448069 |
|  | AC\_000072 |
|  | AAHX01032391 |
|  | AC117950 |
|  | CH473957 |
|  | EDL91541 |
|  | BQ192107 |
|  | CB764502 |
|  | CB793227 |
|  | CB802860 |
|  | CN541827 |
|  | DV727885 |
|  | NC\_018914 |
|  | AMYH02006240 |
|  | XM\_006505669 |
|  | XP\_006505732 |
|  | AC\_000028 |
|  | AAHY01056660 |

---

|  |  |
| --- | --- |
| Swiss-Prot ID | I17RC\_HUMAN |
|  | I17RC\_MOUSE |

---

|  |  |
| --- | --- |
| Cell Localization | Cell membrane |

---

|  |  |
| --- | --- |
| Ensembl ID | ENSG00000163702 |
|  | ENSP00000396064.1 |
|  | ENST00000413608.2 |
|  | ENSP00000395315.3 |
|  | ENST00000416074.6 |
|  | ENSP00000407894.1 |
|  | ENST00000455057.5 |
|  | ENSP00000373323.4 |
|  | ENST00000383812.9 |
|  | ENSP00000384969.3 |
|  | ENST00000403601.8 |
|  | ENSP00000295981.3 |
|  | ENST00000295981.7 |
|  | ENST00000466046.5 |
|  | ENSMUSG00000030281 |
|  | ENSMUSP00000055343.7 |
|  | ENSMUST00000058300.13 |
|  | ENSMUSP00000145038.1 |
|  | ENSMUST00000205208.2 |
|  | ENSRNOG00000027376 |
|  | ENSRNOP00000037747.5 |
|  | ENSRNOT00000037788.6 |
|  | ENSP00000396064 |
|  | ENST00000413608 |
|  | ENSP00000395315 |
|  | ENST00000416074 |
|  | ENSP00000407894 |
|  | ENST00000455057 |
|  | ENSP00000373323 |
|  | ENST00000383812 |
|  | ENSP00000384969 |
|  | ENST00000403601 |
|  | ENSP00000295981 |
|  | ENST00000295981 |
|  | ENST00000466046 |
|  | ENSMUSP00000055343 |
|  | ENSMUST00000058300 |
|  | ENSMUSP00000145038 |
|  | ENSMUST00000205208 |
|  | ENSRNOP00000037747 |
|  | ENSRNOT00000037788 |

---

|  |  |
| --- | --- |
| MGI ID | MGI:2159336 |

---

|  |  |
| --- | --- |
| RGD ID | 1306392 |

---

|  |  |
| --- | --- |
| Unigene ID | Hs.129959 |
|  | Mm.213397 |
|  | Rn.139872 |

---

|  |  |
| --- | --- |
| Homologene ID | 15894 |

---
